# Pharmacokinetic Study of Bioactive Flavonoids in the Traditional Japanese Medicine Keigairengyoto Exerting Antibacterial Effects against *Staphylococcus aureus*

**DOI:** 10.3390/ijms19020328

**Published:** 2018-01-23

**Authors:** Takashi Matsumoto, Atsushi Kaneko, Junichi Koseki, Yosuke Matsubara, Setsuya Aiba, Kenshi Yamasaki

**Affiliations:** 1 Tsumura Kampo Research Laboratories, Kampo Research & Development Division, Tsumura & Co., Ibaraki 300-1192, Japan; kaneko_atsushi@mail.tsumura.co.jp (A.K.); htkj9626m@mineo.jp (J.K.); matsubara_yousuke@mail.tsumura.co.jp (Y.M.); 2 Department of Dermatology, Tohoku University Graduate School of Medicine, Miyagi 980-8574, Japan; saiba@med.tohoku.ac.jp (S.A.); kyamasaki@med.tohoku.ac.jp (K.Y.)

**Keywords:** Keigairengyoto, bacterial infection, phagocytosis, macrophages, *Staphylococcusaureus*, flavonoid, pharmacokinetics

## Abstract

Recent studies have demonstrated that flavonoid glucuronides can be deconjugated to the active form aglycone by β-glucuronidase-expressing macrophages. Keigairengyoto (KRT) is a flavonoid-rich traditional Japanese medicine reported to enhance bacterial clearance through immune modulation. Our aims are to examine the pharmacokinetics of KRT flavonoids and to identify active flavonoids contributing to the adjuvant effects of KRT. KRT was evaluated at pharmacokinetic analysis to quantify absorbed flavonoids, and cutaneous infection assay induced in mice by inoculation of *Staphylococcus aureus*. Preventive or therapeutic KRT administration reduced the number of bacteria in the infection site as well as macroscopic and microscopic lesion scores with efficacies similar to antibiotics. Pharmacokinetic study revealed low plasma levels of flavonoid aglycones after KRT administration; however, plasma concentrations were enhanced markedly by β-glucuronidase treatment, with baicalein the most abundant (*C*_max_, 1.32 µg/mL). In random screening assays, flavonoids such as bacalein, genistein, and apigenin enhanced bacteria phagocytosis by macrophages. Glucuronide bacalin was converted to aglycone baicalein by incubation with living macrophages, macrophage lysate, or skin homogenate. Taken together, the adjuvant effect of KRT may be due to some blood-absorbed flavonoids which enhance macrophage functions in host defense. Flavonoid-rich KRT may be a beneficial treatment for infectious skin inflammation.

## 1. Introduction

Flavonoids are promising bioactive agents against cancer, inflammatory disease, cardiovascular disease, neurological disorders, metabolic syndrome, obesity, and osteoporosis [1,2,3,4], and epidemiological studies show that consumption of flavonoid-rich foods is associated with reduced risks of cancer and cardiovascular disease [5,6]. Moreover, clinical and experimental studies of individual flavonoids such as baicalin, genistein, and tea catechin have reported such efficacies [7,8,9,10,11]. Flavonoids are converted to inactive metabolites like glucuronides in the gut, and circulate mainly as glucuronides in the blood stream, resulting in low concentrations of circulating active aglycones [12,13,14]. It is therefore unclear how oral flavonoids exert bioactivity in tissues, an issue termed “the flavonoid paradox”. 

We and others have demonstrated multiple biological actions of flavonoid glucuronides, and have shown that β-glucuronidase-expressing macrophages convert flavonoid glucuronides into active aglycones, especially at local lesions with inflammation [15,16,17]. Our recent study revealed that genistein 7-*O*-glucuronide activates macrophages and promotes microbe phagocytosis both in vitro and in vivo through deconjugation of the glucuronide and ensuing activation of estrogen signaling [15]. Collectively, these studies have revealed intriguing biological properties of glucuronides as carrier and precursor forms of active flavonoids (aglycones) rather than as simply products of detoxification for excretion. However, detailed studies on the pharmacokinetics and metabolism of flavonoids are needed to understand how glucuronides involve in pathophysiological responses in peripheral tissues.

**Figure 1:**
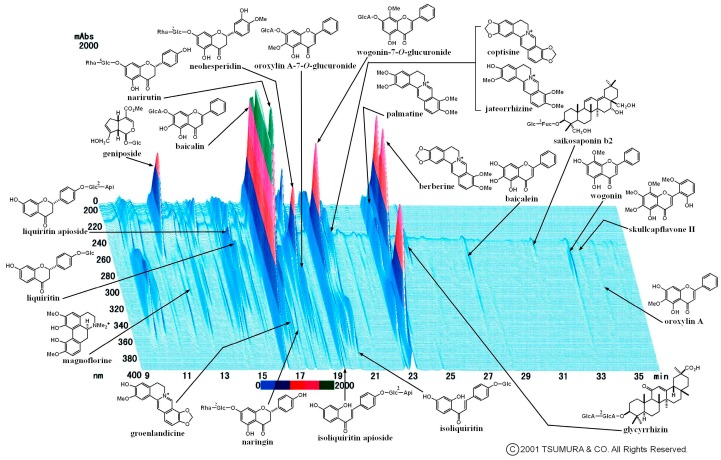
Chemical profile of Keigairengyoto. The chemical profile of Keigairengyoto was analyzed by 3D-HPLC. Each peak in the HPLC profile was identified by comparison of the retention times and UV spectra of chemically defined standard compounds.

Keigairengyoto (KRT) is a pharmaceutical-grade traditional Japanese (kampo) medicine comprised of seventeen crude drugs, and Scutellariae radix, Aurantii fructus immaturus, Schizonepetae spica, and Glycyrrhizae radix especially contains abundant flavonoids. Keigairengyoto is used for several disorders but mainly to treat microbial infection, and is also prescribed for purulent inflammation such as dermatoses, empyema, and rhinitis. The three-dimensional high-performance liquid chromatograph (3D-HPLC) profile of KRT provided by Tsumura & Co., shows that KRT includes various compounds, especially flavonoids, triterpenoids, and isoquinoline alkaloids (Figure 1). We previously reported that oral administration of KRT suppressed microbe-induced dermatosis in mice and enhanced bacterial clearance through modulation of the immune system [18]. It is likely that bioactive flavonoids derived from KRT mediate the adjuvanticity against microbes. However, it is unclear which specific flavonoids are absorbed in plasma after oral administration of KRT and contribute to these antibacterial effects.

The flavonoid glucuronide is one possible mediator of KRT efficacy. Our aims in this study are to examine the plasma pharmacokinetics of KRT flavanoid-derived aglycones and glucuronides and to identify active flavonoids contributing to the antibacterial efficacy of KRT. 

## 2. Results

### 2.1. KRT Exhibited Antibacterial Effects in Cutaneous Infection

**Figure 2:**
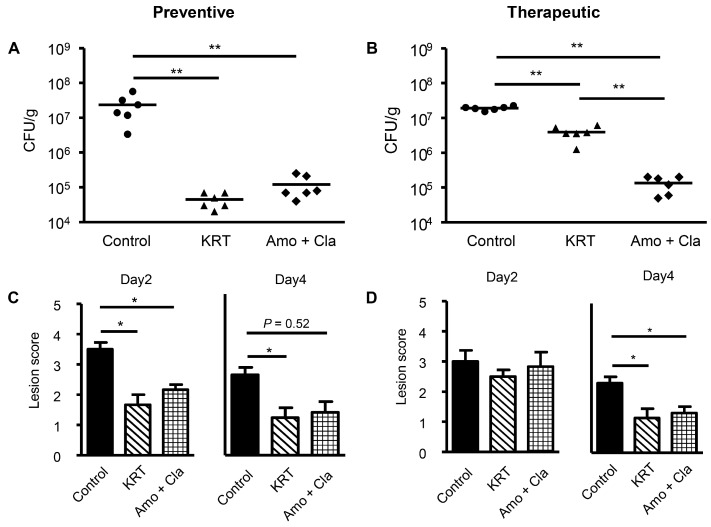
Keigairengyoto demonstrated antibacterial activity against superficial skin *Staphylococcus aureus* infection comparable to antibiotic drugs. After tape stripping the back skin of mice, *S. aureus* was inoculated at 1 × 10^7^ CFU. Keigairengyoto (KRT, 2 g/kg), antibiotic (amoxicillin 100 mg/kg and clavulanic acid 50 mg/kg), or water (vehicle) was administered orally to mice 1 h before and one, two, and three days after inoculation (preventive administration regimen), or one, two, and three days after inoculation (therapeutic administration). Back skin was excised on day 4 for measurement of living bacteria (**A**: preventive protocol groups, **B**: therapeutic protocol groups). Macroscopic damage scores at infection sites were evaluated on day 2 and 4 (**C**: preventive, **D**: therapeutic). Each value represents the mean ± SEM. *n* = 6 vehicle control, KRT, and antibiotic group mice. **: *p* < 0.01; *: *p* < 0.05 (Steel-Dwass test).

**Figure 3:**
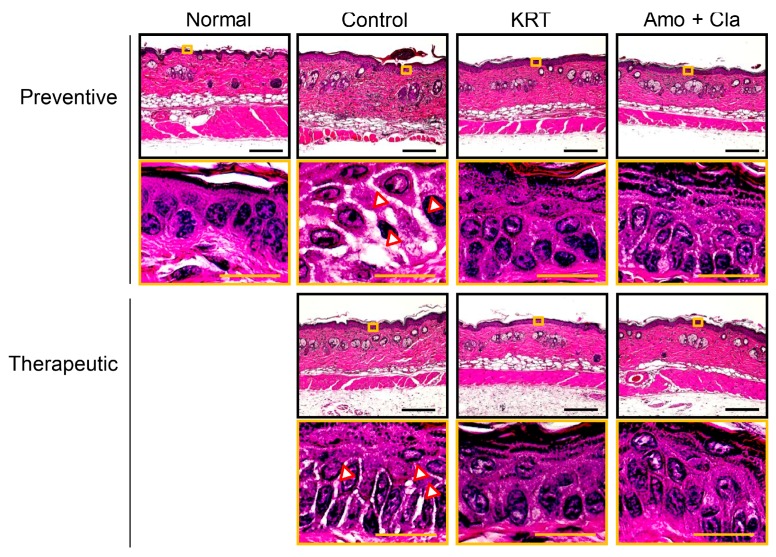
Representative images of superficial skin infection by *Staphylococcus aureus*. Bacterial infection and test sample administrations were performed under the protocols described in Figure 2. Skin sections excised on day 4 were stained with hematoxylin and eosin. Representative images are shown at low magnification (upper panels) and high magnification (lower panels). Yellow boxes demarcate sites of magnification and arrows show sites of spongiosis. Black bars: 200 μm. Yellow bars: 50 μm.

**Figure 4:**
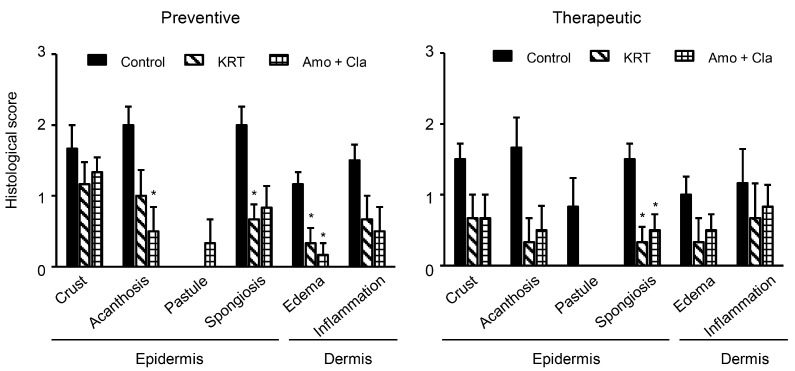
Keigairengyoto suppressed microscopic changes induced by superficial skin infection by *Staphylococcus aureus*. Bacterial infection and test sample administration were performed under the protocols described in Figure 2. The skin sections on day 4 were stained with hematoxylin and eosin. Histological findings in epidermis and dermis were scored according to the following criteria: 0 (none), 1 (slight), 2 (mild), 3 (moderate), and 4 (marked). Each value represents the mean ± SEM. *n* = 6 vehicle control, KRT, and antibiotic group mice. *: *p* < 0.05 (Steel’s test).

We investigated the antibacterial activity of KRT in superficial skin infection from local *S. aureus* inoculation by comparing the number of surviving bacteria in lesions among vehicle-treated control mice, antibiotic-treated mice (100 mg/kg amoxicillin plus 50 mg/kg clavulanic acid), and mice receiving KRT (2 g/kg/day). Treatments were delivered by two administration regimens, preventive (treatment starting 1 h before inoculation) or therapeutic (starting one day after inoculation). The mean number of living bacteria at four days post-inoculation was markedly reduced by preventive administration of KRT compared to untreated controls (0.5 ± 0.1 × 10^5^ vs. 235.9 ± 77.7 × 10^5^ colony forming units, CFU/g) (Figure 2A). In fact, the antibacterial activity of preventive treatment was similar to preventive treatment with antibiotic drugs (1.2 ± 0.4 × 10^5^ CFU/g). Macroscopic lesion scores at two and four days post-inoculation were lower in KRT-treated and antibiotic-treated mice than control mice (Figure 2C). Therapeutic administration of KRT also significantly reduced the number of living *S. aureus* in the cutaneous infection, although the action was weaker than that in the therapeutic antibiotic group (Figure 2B). However, suppression of skin lesion scores by therapeutic administration of KRT was similar to antibiotic drugs (Figure 2D). Histological observation revealed that KRT (preventive and therapeutic) suppressed spongiosis in epidermis and edema in dermis (Figure 3). Quantitative analyses are shown in Figure 4.

### 2.2. Multiple Flavonoids and Glucuronide Metabolites Were Identified in Plasma of KRT-Administered Rats

**Figure 5:**
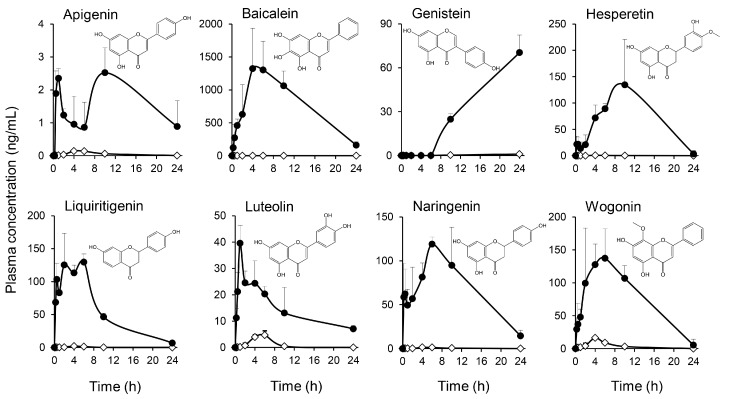
Multiple flavonoids derived from Keigairengyoto quantified in plasma before and after enzymatic hydrolysis with β-glucuronidase. Keigairengyoto (KRT) was given orally to rats at 2 g/10 mL/kg. Plasma samples were obtained 0.25, 0.5, 1, 2, 4, 6, 10, or 24 h after KRT administration. To confirm the presence of glucuronides, the collected plasma samples were incubated with β-glucuronidase for 2 h at 37 °C. The plasma concentrations were measured by liquid chromatography–mass spectrometry with tandem mass spectrometry before (white diamonds) and after (black circles) β-glucuronidase treatment. Each data point represents the mean ± S.D. of triplicate measurements.

As shown in Figure 1, KRT includes various compounds, especially flavonoids, triterpenoids, and isoquinoline alkaloids. We selected eight flavonoids represented in Table 1, glycyrrhizic acid, glycyrrhetinic acid and berberine, and conducted their pharmacokinetic study using the plasma of rats given orally with 2 g/kg KRT. Figure 5 and Table 1 show the plasma concentration−time profiles and pharmacokinetic parameters of eight flavonoid aglycones before and after β-glucuronidase treatment. The concentrations of flavonoids aglycone, apigenin, baicalein, genistein, hesperetin, liquiritigenin, luteolin, naringenin, and wogonin were very low or below the quantification limits (BQL) in plasma samples without β-glucuronidase treatment. However, β-glucuronidase treatment increased concentrations of almost all flavonoids (e.g., maximum concentration of baicalein was 1320 ng/mL after β-glucuronidase treatment, while the concentration was BQL in untreated plasma from KRT-administered rats). This study revealed that glucuronides of baicalein, wogonin, liquiritigenin, and genistein were largely increased in plasma from 2 to 10 h after KRT administration. 

Glycyrrhizic acid was found in plasma of rats given with KRT, showing the maximum concentration (*C*_max_): 2.88 ng/mL at 1 h after the KRT administration, while the main metabolite glycyrrhetinic acid was found showing *C*_max_: 121 ng/mL at 6 h after the KRT administration. On the other hand, concentrations of berberine were BQL 0.181 ng/mL all time points no matter whether the plasma was treated with β-glucuronidase.

**Figure 6:**
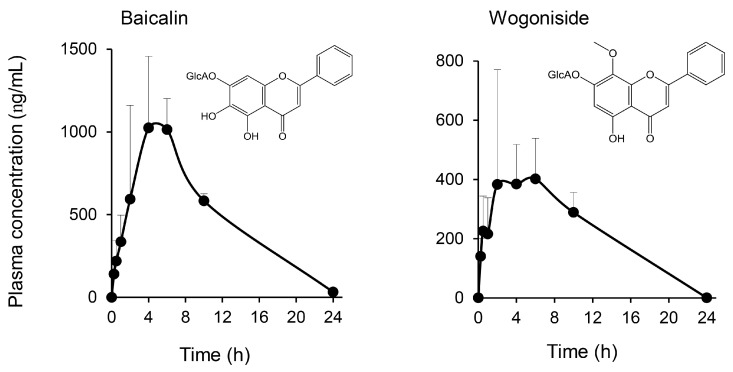
Flavonoid glucuronide plasma concentrations quantified using authentic compounds. Plasma samples from rats administered KRT were harvested under the protocol described in Figure 5. Baicalin and wogonoside were measured in plasma by liquid chromatography–mass spectrometry with tandem mass spectrometry. Each data point represents the mean ± S.D. of triplicate measurements. GlcA: glucuronic acid.

We also measured plasma concentrations of baicalin and wogonoside, the representative glucuronide forms of baicalein and wogonin, respectively, using authentic compounds. As shown in Figure 6, both glucuronides were detected in untreated plasma from KRT-administered rats. The pharmacokinetic parameters of baicalin and wogonoside were as follows: time to maximum concentration (*t*_max_), 4 and 6 h; *C*_max_, 1.02 and 0.402 µg/mL; area under the plasma concentration-time curve from time zero to the last observation (AUC_0–*last*_), 10.1 and 3.40 µg/mL; apparent elimination half-life (*t*_1/2_), 3.53 h and not calculated, respectively.

### 2.3. Active Flavonoids of KRT Augmented Phagocytosis by Macrophages

According to recent studies by us and others, resident macrophages in tissue express β-glucuronidase and can deconjugate flavonoid glucuronides to bioactive aglycones. To identify flavonoids that may contribute to the antibacterial efficacy of KRT, we tested the capacities of various active flavonoid aglycones to promote phagocytosis by macrophages in vitro. Apigenin, baicalein, genistein, liquiritigenin, luteolin, naringenin, and wogonin enhanced phagocytosis by macrophages at 10 μmol/L, while hesperetin did not (Table 2). Apigenin, baicalein, and genistein in particular showed robust phagocytosis-promoting activities (3.3, 3.4, and 3.4 times vehicle control, respectively). We also directly compared the phagocytosis-promoting activity of baicalein to its glucuronide form baicalin. Glucuronide baicalin at 10 μmol/L promoted phagocytosis by macrophages, although the activity was weaker than aglycone baicalein at 3 and 10 μmol/L (Table 2, Exp. 2).

### 2.4. Macrophages Converted Flavonoid Glucuronide Baicalin to Aglycone Baicalein

**Figure 7:**
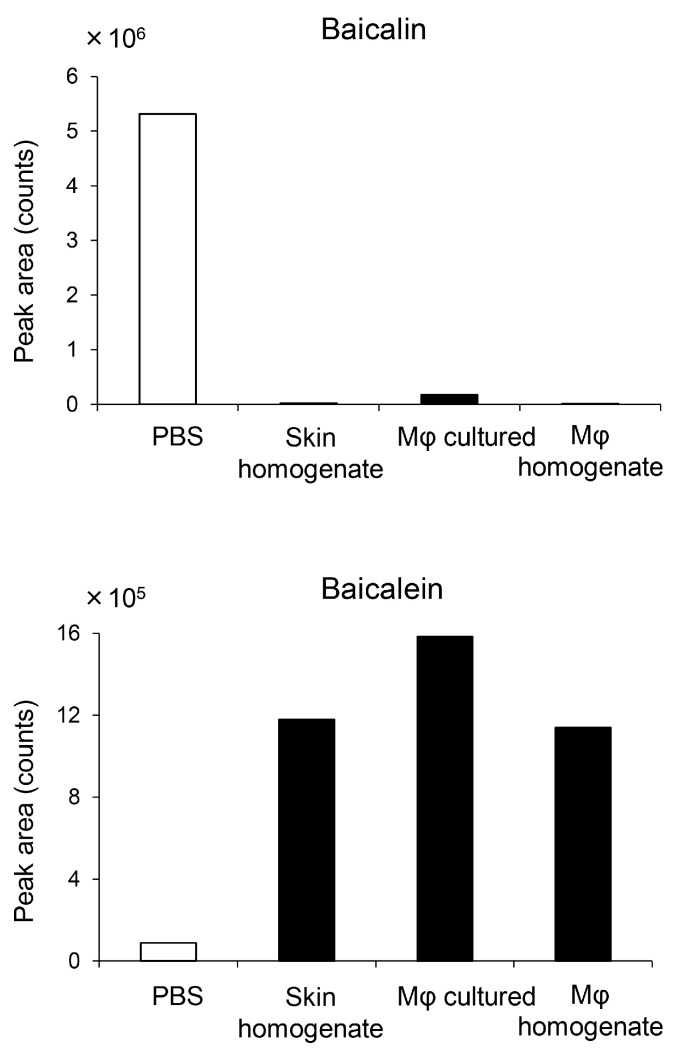
Capacity of skin-resident macrophages to deconjugate baicalin to aglycone baicalein. Baicalin was added at 10 μmol/L to the following biosources of β-glucuronidase in 24 well plates: homogenates of back skin from normal mice (skin homogenate), live RAW264.7 cells seeded at 1 × 10^5^ cells/0.5 mL/well (Mφ cultured), and RAW264.7 cell lysates (Mφ homogenate). After 24 h incubation in a 5% CO_2_ incubator, baicalin and baicalein were quantified by liquid chromatography–mass spectrometry with tandem mass spectrometry. Detection limits: 0.00224 μmol/L for baicalin, 0.0370 μmol/L for baicalein. Each data point represents the mean of duplicate measurements.

It was reported that macrophages express functional β-glucuronidase; we therefore tested for flavonoid glucuronide-to-aglycone conversion by cultured macrophages and homogenates of skin. As shown in Figure 7, incubation with live macrophages or homogenized lysates converted baicalin (10 μmol/L starting concentration) to aglycone baicalein. Skin homogenate showed similar conversion capacity. These results confirm that macrophages, including resident skin macrophages, possess functional β-glucuronidase that converts baicalin to aglycone baicalein.

## 3. Discussion

### 3.1. Antibacterial Activity of KRT

The number of living *S. aureus* was significantly reduced by both preventive and therapeutic administration of KRT. Two possible mechanisms may underlie the substantial antimicrobial activity of KRT, direct bactericide and immune modulation. Bactericidal ingredients derived from KRT may directly attack bacteria. Indeed, KRT was reported to have direct antibacterial activity against *S. aureus* and *Propionibacterium acnes* in vitro [19,20]. Further, flavonoids contained in KRT, including baicalein and wogonin, have bactericidal effects [21,22]. Second, KRT may exert antimicrobial effects by enhancing immune system activity, such as macrophage phagocytosis. We previously examined the effects of KRT in a pseudo-infection model using heat-killed *S. aureus* in order to eliminate the possibility of direct bactericidal effects and observed augmented bacteria clearance as evidenced by biochemical, histological, and immunological methods [18]. Moreover, macrophage activation was promoted by treatment with phytoestrogen flavonoids [23,24,25,26,27,28,29]. Therefore, the antimicrobial effects of KRT are likely mediated indirectly via promotion of macrophage functions, particularly phagocytosis. Moreover, these results implicate flavonoids as key bioactive compounds contributing to the bactericidal activity of KRT.

### 3.2. Pharmacokinetics of Multiple Flavonoids of KRT

Since KRT is an oral preparation, it is critical to quantify the ingredients actually reaching the blood to elucidate the underlying mechanisms against skin infection. All flavonoids examined except genistein showed a bimodal or trimodal plasma concentration after KRT administration. In contrast, genistein concentration was below the detection limit in β-glucuronidase-treated plasma until 6 h, and was then detected at 10 and 24 h after KRT administration. Although it is unclear why the profile of genistein differed from the other flavonoids, distinct metabolic pathways among these compounds is possible given that flavonoids were subject to various metabolic pathways in living body [30]. Alternatively, an unknown precursor of genistein in KRT might be involved. As evidenced in previous studies [31], plasma concentration-time profiles also depend on the gut site where each flavonoid is absorbed and/or metabolized. The small and large intestines have different functions and harbor distinct microflora, which influences the metabolism of flavonoids. Further studies are necessary to clarify the metabolic pathways for specific KRT flavonoids.

The tissue distribution of polyphenols have been reported for oral administration, including distribution to skin [32,33,34]. After administration of [3H](−)-epigallocatechin gallate to mouse stomachs, radioactivity was detected in various tissues, including the liver, kidney, lung, brain, and skin [34], suggesting a wide tissue distribution of polyphenols after absorption. Therefore, KRT flavonoids were considered to migrate to various tissues, including the skin, after oral administration and exert their pharmacological action.

### 3.3. Baicalin/Baicalein Is the Main Flavonoid Contributing to the Antibacterial Activity of KRT and Macrophages Are Involved in Deconjugation and Activation to Aglycone Baicalein

The present study also revealed a higher concentration of glucuronide baicalin in the systemic circulation compared to the other flavonoids measured following oral KRT (Figure 5 and Figure 6, and Table 1). Since flavonoids circulate predominantly as glucuronides rather than as active aglycones, it is reasonable to assume that activation occurs locally at sites of inflammation. Skin-resident macrophages function as sentinels and as the first line of defense against microbes together with other innate immune cells such as dendritic and Langerhans cells. In a random screening assay, baicalein strongly enhanced phagocytosis by macrophages. The glucuronide baicalin also exhibited this activity, suggesting conversion in macrophages. This may be due to macrophage β-glucuronidase because baicalin was almost completely deconjugated to baicalein during 24 h incubation in the presence of living macrophages (Figure 7) as well as lysates of both macrophages and skin. It is therefore plausible that resident macrophages convert bacalin to baicalein in skin and that baicalin/baicalein are the main flavonoids contributing to the antibacterial activity of KRT. 

Recent papers have demonstrated promising profiles of baicalin as an anti-microbe and anti-inflammatory agent. For instance, baicalin induces the activation of autophagy on the *Mycobacterium tuberculosis*-infected macrophages through PI3K/Akt/mTOR pathway [35]. Moreover, baicalin is reported to inhibit NOD-like receptor (NLR) family, pyrin containing domain 3 (NLRP3) inflammasome activation partly through augmenting PKA signaling, and improve survival in an animal infection model using *Escherichia coli* [36]. 

Glycyrrhizic acid and berberine are bioactive compounds found abundantly in the KRT extract (Figure 1). We measured their levels in the plasma of KRT-treated rats, but the concentrations of both compounds were extremely low or BQL. Glycyrrhetinic acid, the main metabolite of glycyrrhizic acid, was detected in the plasma after KRT administration. Our previous data showed that glycyrrhetinic acid has no macrophage phagocytosis-enhancing activity [18]. However, many pharmacological effects have been reported for glycyrrhetinic acid, including anti-inflammatory [37], anti-allergic [38], and analgesic effects [39], suggesting that glycyrrhetinic acid contributes to KRT efficacies.

### 3.4. Comparison with Our Previous Papers Showing Genistein 7-O-glucuronide as a Key Flavonoid for Bacterial Clearance

In our previous studies, we examined three flavonoid aglycones, genistein, liquiritigenin, and hesperetin, based on the results of pharmacokinetic studies of flavonoids detected in animals orally administered another kampo medicine, Jumihaidokuto [14]. We also reported that intravenous injection of genistein 7-*O*-glucuronide promoted macrophage activity in the inflamed skin of a microbial infection model, and identified aglycone genistein in the inflamed skin in addition to the injected glucuronide [15]. Moreover, macrophage culture assays revealed that genistein 7-*O*-glucuronide increased phagocytosis, and that both β-glucuronidase and estrogen receptor signaling in macrophages were involved in this activation. Since genistein, liquiritigenin, and their glucuronides also appeared in the plasma of KRT-administered rats, these may be active ingredients in KRT as in Jumihaidokuto [18]. However, KRT includes more abundant flavonoid-rich crude drugs than Jumihaidokuto, such as Scutellariae radix (baicalin, baicalein, wogonin), Aurantii fructus immaturus (naringenin, hesperetin), Schizonepetae spica (apigenin, hesperetin, luteolin), and Glycyrrhizae radix (genistein, liquiritigenin). The complex mixture of flavonoids and other ingredient can affect absorbance and metabolism of flavonoids, so we examined which flavonoid actually appeared in plasma following KRT administration. The present study revealed seven flavonoid aglycones, including genistein and liquiritigenin, in plasma of KRT-administered rats and demonstrated that each enhanced phagocytosis by macrophages. These seven flavonoids were all reported to bind estrogen receptors and/or interact with estrogen receptor signaling [40,41]. A docking simulation between bacalein and human estrogen receptor was also reported [42]. Estrogen has been reported to modulate the differentiation, maturation, lifespan, and effector functions of innate immune cells, including neutrophils, macrophages, natural killer cells, and dendritic cells [43]. These lines of evidence suggest that flavonoid-rich KRT exerts antibacterial effects through activation of macrophage functions in host defense, including phagocytosis. 

### 3.5. Possible Utility of KRT as a Complement to Antibiotics

Antibiotics are beneficial therapeutic drugs for skin infections. However, inappropriate applications such as long-term administration has resulted in the emergence of resistant bacteria [44]. Moreover, long-term administration can alter the gut microbiome [45], which may contribute to side effects such as constipation. The World Health Organization has for the first time released a list of drug-resistant bacteria that pose the greatest threat to human health [46], and methicillin- and/or vancomycin-resistant *S. aureus* are listed as among the most dangerous strains. It is noteworthy that the species used in the present study was methicillin-resistant *S. aureus* (provided by the ATCC). Preventive administration of KRT drastically reduced the number of *S. aureus* at the infection site and both macroscopic and microscopic lesion scores. These antimicrobial effects of KRT were also observed in response to therapeutic administration. The antimicrobial efficacy of KRT is therefore of potential clinical significance, and we suggest that KRT may be beneficial for adjunct treatment of infectious skin diseases.

## 4. Materials and Methods 

### 4.1. Test Drugs

Keigairengyoto was supplied by Tsumura & Co. (lot number 2140050010, Tokyo, Japan) as a powdered extract obtained by spray-drying a hot water extract mixture of the following seventeen crude drugs: Scutellariae radix, Phellodendri cortex, Coptidis rhizoma, Platycodi radix, Aurantii fructus immaturus, Schizonepetae spica, Bupleuri radix, Gardeniae fructus, Rehmanniae radix, Paeoniae radix, Cnidii rhizoma, Angelicae radix, Menthae herba, Angelicae dahuricae radix, Saposhnikoviae radix, Forsythiae fructus, and Glycyrrhizae radix.

Amoxicillin and clavulanic acid used as reference antimicrobial drugs were purchased from Toronto Research Chemicals (Toronto, ON, Canada). Pure samples of apigenin, baicalein, baicalin, genistein, hesperidin, hesperetin, liquiritin, liquiritigenin, naringenin, glycyrrhizic acid, and glycyrrhetinic acid were purchased from Wako Pure Chemical Industries (Osaka, Japan). Apigenin 7-*O*-glucoside was purchased from Sigma-Aldrich (St. Louis, MO, USA), narirutin from Extrasynthese (Genay, France), and isoliquiritin from Chemfaces (Hubei, China). Baicalein, baicalin (baicalein 7-*O*-glucuronide), luteolin, genistin, wogonin, and wogonoside (wogonin 7-*O*-glucuronide), and berberine chloride were obtained from Tsumura & Co., with high purities for pharmacokinetic study.

### 4.2. Animals

Male Sprague–Dawley rats were purchased from Charles River Laboratories (Yokohama, Japan) and used at 8 week of age for pharmacokinetic study. Male BALB/c mice were purchased from Japan SLC (Shizuoka, Japan) and used at 7 weeks old. All studies were approved by and conducted according to the guidelines of the experimental animal ethics committees of Tsumura & Co. (permit no.: 16-072 (19 December 2016) and 17-002 (5 April 2017)). All surgeries were performed under ketamine or isoflurane anesthesia, and all efforts were made to minimize suffering. In the present study, KRT was administrated orally to the experimental animals at 2 g/kg in distilled water. Although the dose of KRT was higher than the clinical dose used in humans, this dose was based on previous publications of experimental KRT studies [18] reporting the beneficial effects of KRT related to clinical efficacy. No adverse effects were observed in animals given 2 g/kg KRT in the present experiment.

### 4.3. Superficial Skin Infection Model

Cutaneous infection by S. aureus was induced in BALB/c mice weighting 17.9 to 21.8 g according to the procedure described by Kugelberg et al. with a minor modification [47]. Forty mice were randomly divided into seven groups as follows: normal (n = 4) non-infection group, vehicle control (n = 6 × 2), KRT-treated (n = 6 × 2), and antibiotics-treated (n = 6 × 2) infection groups. The hair on the back was shaved with an electric razor and depilatory cream applied under ketamine anesthesia. An area of skin approximately 4 cm^2^ was stripped with clothmade cohesive tape once a day for two days. After stripping on the second day, S. aureus MW2 (strain: BAA-1707, ATCC, Manassas, VA, USA) was inoculated on the skin at 1 × 10^7^ CFU in 100 μL saline. Keigairengyoto was administrated orally at 2 g/kg in distilled water 1 h before and 1, 2, and 3 days after the S. aureus inoculation (preventive administration) or 1, 2, and 3 days after inoculation (therapeutic administration). As reference drugs, a mixture of amoxicillin (100 mg/kg) and clavulanic acid (50 mg/kg) was prepared in water and administrated orally following the same schedules as for KRT. The macroscopic evaluation of skin lesion was examined at days 2 and 4 after the inoculation. The histological evaluation and the count of living S. aureus on the skin were performed at day 4 after the inoculation.

Living *S. aureus* on the skin were counted 4 days after inoculation with *S. aureus*, according to a previous paper [19]. Immediately after the mice were killed, the wounds, approximately 2 cm^2^, were excised and homogenized together with 1 mL of phosphate-buffered saline in Stomacher lab system bags using a Stomacher 80 machine (Seward Ltd., Thetford, UK) set at 260 strokes per min for 120 s. Suitable dilutions of the homogenates were plated on *S. aureus* agar plates to determine the number of living bacteria (CFU). The number of living bacteria is reported as CFU per weight of the skin tissues (CFU/g).

The macroscopic evaluation was performed 2 and 4 days after inoculation with *S. aureus*. The severities of erosion and the papule were evaluated according to a previously published report with a minor modification [48]: 0 (none), 1 (mild), 2 (moderate), and 3 (severe), and are reported as the sum of each score. The observer was blinded to the treatments for all animals. 

To characterize the histopathology, biopsy specimens were taken 4 days after inoculation with *S. aureus*. Immediately after the animals were killed, 5-mm punch biopsy specimens of excised skin were fixed in phosphate-buffered (pH 7.4) formalin (4%). The formalin-fixed biopsy specimens were embedded in paraffin and stained with hematoxylin and eosin. The histological findings of the crust, acanthosis, pastule and spongiosis in the epidermis, and edema and inflammation in the dermis were scored according to the following criteria, respectively: 0 (none), 1 (slight, <25% in full field), 2 (mild, 25–50%), 3 (moderate, 50–75%), and 4 (marked, >75%). The observer was blinded to the treatments for all biopsy specimens.

### 4.4. Pharmacokinetic Analysis of KRT-Derived Flavonoids and Glucuronide Metabolites by Liquid Chromatography–Mass Spectrometry with Tandem Mass Spectrometry

Keigairengyoto prepared in water was given orally to 16-h fasted rats (weighing 253 to 273 g) at a dose of 2 g/10 mL/kg. Blood was withdrawn from the abdominal inferior vena cava with a heparinized syringe at 0.25, 0.5, 1, 2, 4, 6, 10, or 24 h after administration (*n* = 3). Plasma was obtained by centrifugation at 1700× *g* for 15 min at 4 °C and stored at −75 °C or colder until analysis. To specifically deconjugate glucuronides, 80 μL of plasma sample was incubated with 50 μL of β-glucuronidase solution containing 200 units/mL β-glucuronidase from *Escherichia coli* (Sigma-Aldrich) for 2 h at 37 °C. The deconjugation reaction was stopped by adding ethyl acetate or by refrigerating the mixture considering the following procedures. This pretreated solution was injected into the two liquid chromatography–mass spectrometry with tandem mass spectrometry (LC-MS/MS) systems after pooling with an internal standard solution of niflumic acid (Sigma-Aldrich) or atropine (Wako Pure Chemical Industries, Osaka, Japan). Analytical conditions are summarized in Tables S1 and S2.

For quantification of apigenin, wogonin, wogonoside, hesperetin, naringenin, liquiritigenin, genistein, and luteolin in β-glucuronidase-naive (untreated) plasma, 200 µL plasma samples were pretreated by liquid-liquid extraction using ethyl acetate. The total supernatant was collected, dried, and concentrated using a centrifugal evaporator. The dried residue was then dissolved in 60 μL of the specific HPLC mobile phase used for each analytical method, and a 10 or 20 μL volume injected into the LC-MS/MS system.

For quantification of baicalin, baicalein, and berberine in untreated plasma, 80 µL of plasma was pretreated by solid-phase extraction using Oasis HLB 96-well µElution plate (Waters, Milford, MA, USA). The methanol eluates were collected, dried, and dissolved in 60 μL of the specific HPLC mobile phase. A 10 or 20 μL volume was then injected into the LC-MS/MS system.

For quantification of glycyrrhizic acid and glycyrrhetinic acid in untreated plasma, 100 µL plasma samples were pretreated by deproteinization using methanol. The supernatant was collected, dried, and concentrated using a centrifugal evaporator. The dried residue was then dissolved in 60 μL of the specific HPLC mobile phase used for the analytical method, and a 10 μL volume injected into the LC-MS/MS system.

For quantification of wogonin, wogonoside, hesperetin, naringenin, liquiritigenin, and genistein in treated plasma, the reaction solutions after deconjugation reactions were pretreated by liquid-liquid extraction. The supernatant was dissolved in 120 μL of the specific HPLC mobile phase, and a 10 or 15 μL volume injected into the LC-MS/MS system.

For quantification of apigenin, luteolin, baicalin, baicalein, and berberine in treated plasma, the reaction solutions after deconjugation were pretreated by solid-phase extraction by the method described above, and 10 or 15 μL of the solution dissolved in 50 μL of the specific HPLC mobile phase was injected into the LC-MS/MS system.

Pharmacokinetic parameters of flavonoids and glucuronide metabolites in KRT-administrated plasma with or without β-glucuronidase treatment were analyzed by noncompartmental modeling using Phoenix WinNonlin (version 6.3, Certara L.P., St. Louis, MO, USA) to determine various pharmacokinetic constants, including *C*_max_, *t*_max_, *t*_1/2_, and AUC_0–*last*_. The *t*_1/2_ was divided by loge2/*ke*, where *ke* is the terminal elimination rate constant determined from at least three data points on the descending linear limb.

### 4.5. Macrophage Culture Assay

Cells of the mouse macrophage cell line RAW264.7 (ATCC, Manassas, VA, USA) were grown in Dulbecco’s modified Eagles medium supplemented with 10% fetal bovine serum (FBS), 4.5 g/L glucose, 2 mmol/L l-glutamine, 100 U/mL penicillin, 100 µg/mL streptomycin, and 10 mmol/L HEPES. Cells were seeded in 96-well culture plates at 5 × 10^3^ cells/well and cultured with the test compound (30 μmol/L) in the presence or absence of a suboptimal dose (0.5 ng/mL) of mouse interferon-γ (IFN-γ) (PeproTech, Rocky Hill, NJ, USA). After 3 days’ incubation at 37 °C, culture fluids were removed and replaced by warm medium containing 30 μg/mL FITC-conjugated *S. aureus*. After 30 min incubation in a 5% CO_2_ incubator, cells were harvested using cold PBS containing 2 mmol/L EDTA, washed, and fixed for 15 min at 4 °C with phosphate buffer containing 4% paraformaldehyde (pH 7.4). FITC-positive cells were determined using a FACSaria II flow cytometer and DIVA 8.0.1 software (Becton Dickinson, San Jose, CA, USA). Phagocytic activity is expressed as the mean fluorescence intensity (MFI) from individual cells.

### 4.6. Conversion Assay of Baicalin to Baicalein

Naïve mice were sacrificed by exsanguination to obtain skin samples from the back. After shaving with an electric razor, the skin was excised and homogenized in cold PBS at 20 mg/mL. After centrifugation at 10,000× *g* for 20 min at 4 °C, supernatants were harvested and stored at −80 °C until use. 

To examine β-glucuronidase activity in skin tissue, baicalin (final 10 μmol/L) was added as a substrate to skin homogenate in 24-well plates. Alternatively, to examine β-glucuronidase activity in macrophages, baicalin (final 10 μmol/L) was added to live RAW264.7 cells seeded at 1 × 10^5^ cells/0.5 mL/well or to cell lysates in 24-well plates. After 24 h incubation in a 5% CO_2_ incubator, supernatants were harvested and stored at −80 °C until measurement of baicalin and baicalein by LC-MS/MS as described above.

### 4.7. Statistical Analysis

Paired group means were compared by Student’s *t*-test and multiple group means by Steel, Steel-Dwass, or Dunnett test. For all tests, a probability of less than 0.05 (2-tailed) was considered significant.

## 5. Conclusions

Our study yielded two important findings. First, we verify the antimicrobial activity of KRT under both preventive and therapeutic administration protocols. The possible clinical efficacy of KRT is noteworthy given the emphasis on reduced antibiotic administration to limit antimicrobial resistance. Baicalin is one of the key flavonoids mediating this antimicrobial effect. Second, we describe the pharmacokinetics of flavonoids present in KRT and provide a possible explanation for what we and others have termed the “flavonoid paradox” regarding oral flavonoids. Specifically, flavonoids circulate as inactive glucuronides and are converted to active aglycones at local inflammatory lesions, possibly by β-glucuronidase-expressing macrophages. The pharmacokinetics and bioactive functions of individual glucuronides should be studied in greater detail to further refine flavonoid-based treatments.

## Figures and Tables

**Table 1:** Pharmacokinetic parameters of flavonoids quantified in plasma of Keigairengyoto-administered rats.

Treatment	Compound	*t*_max_ (h)	*C*_max_ (ng/mL)	AUC_0–*last*_ (ng·h/mL)	*t*_1/2_ (h)
No treatment	Apigenin	4.00	0.140	0.843	-
	Baicalein	-	BQL ^#^	-	-
	Genistein	24.0	0.998	9.65	-
	Hesperetin	4.00	0.644	2.83	-
	Liquiritigenin	4.00	2.11	8.68	-
	Luteolin	6.00	4.70	21.3	-
	Naringenin	4.00	1.34	9.53	3.01
	Wogonin	4.00	16.3	71.5	-
Treatment with β-glucuronidase	Apigenin	10.0	2.52	35.7	-
	Baicalein	4.00	1320	16,800	5.72
	Genistein	24.0	75.2	763	-
	Hesperetin	10.0	135	735	-
	Liquiritigenin	6.00	130	1270	-
	Luteolin	1.00	39.6	348	11.7
	Naringenin	6.00	119	1470	-
	Wogonin	6.00	146	1150	-

The collected plasma samples were incubated with β-glucuronidase for 2 h at 37 °C. The concentrations of flavonoids in plasma samples of Keigairengyoto-given rats were quantified by liquid chromatography–mass spectrometry with tandem mass spectrometry before and after β-glucuronidase treatment. Pharmacokinetic parameters of flavonoids were analyzed by noncompartmental modeling using Phoenix WinNonlin (version 6.3, Certara L.P., St. Louis, MO, USA) to determine various pharmacokinetic constants. ^#^: Baicalein concentrations in plasma with no treatment were below the quantification limit (BQL, 125 ng/mL) at all time points. *C*_max_: maximum concentration, *t*_max_: time to maximum concentration, *t*_1/2_: apparent elimination half-life, AUC_0–*last*_: area under the plasma concentration-time curve from time zero to the last observation, -: Not calculated.

**Table 2:** Effects of Keigairengyoto-related flavonoids on macrophage phagocytosis.

Experiment No.	IFN-γ	Test Sample	Concentration (μmol/L)	Phagocytosis MFI (Relative Change ^#^)
Exp. 1	−	No IFN-γ	-	144 ± 6
	+	Control	-	155 ± 4
	+	apigenin	10	507 ± 11 ** (3.3)
	+	baicalein	10	530 ± 6 ** (3.4)
	+	genistein	10	525 ± 22 ** (3.4)
	+	hesperetin	10	193 ± 12 (1.2)
	+	liquiritigenin	10	245 ± 4 ** (1.6)
	+	luteolin	10	408 ± 22 ** (2.6)
	+	naringenin	10	236 ± 10 ** (1.5)
	+	wogonin	10	271 ± 16 ** (1.7)
Exp. 2	−	No IFN-γ	-	162 ± 1
	+	Control	-	226 ± 12
	+	baicalein	1	223 ± 10 (1.0)
	+	baicalein	3	445 ± 7 ** (2.0)
	+	baicalein	10	866 ± 25 ** (3.8)
	+	baicalin	1	238 ± 9 (1.1)
	+	baicalin	3	269 ± 10 (1.2)
	+	baicalin	10	531 ± 19 ** (2.4)

Mouse macrophage-like RAW264.7 cells were seeded in 96-well culture plates at 5 × 10^3^ cells/well and cultured with test flavonoids (concentration in Exp. 1: 10 μmol/L; concentrations in Exp. 2: 1, 3, and 10 μmol/L) in the presence or absence of a suboptimal dose of mouse interferon-γ (IFN-γ, 0.5 ng/mL). The culture fluids were removed after 3 days and replaced with fresh medium containing FITC-conjugated *S. aureus* (30 μg/mL), followed by an additional incubation for 0.5 h. Cells were harvested, and FITC-positive cells measured by FACSaria II. The screening assay Exp. 1 and concentration-dependent assay Exp. 2 were conducted three times, respectively. The representative results are shown as mean ± SEM of triplicates. MFI: mean fluorescence intensity. **: *p* < 0.01 vs. IFN-γ alone control (Dunnett’s test). ^#^: relative change versus control.

## Supplementary Materials

Supplementary materials can be found at http://www.mdpi.com/1422-0067/19/2/328/s1.

## Abbreviations

KRT	Keigairengyoto
3D-HPLC	Three-dimensional high-performance liquid chromatograph
CFU	Colony forming units
BQL	Below the quantification limit (BQL)
*C*_max_	Maximum concentration
*t*_max_	Time to maximum concentration
*t*_1/2_	Apparent elimination half-life
AUC_0–*last*_	Area under the plasma concentration-time curve from time zero to the last observation
MFI	Mean fluorescence intensity
LC-MS/MS	Liquid chromatography–mass spectrometry with tandem mass spectrometry
FBS	Fetal bovine serum
