# Supplementary material for: Pharmacokinetic Study of Bioactive Flavonoids in the Traditional Japanese Medicine Keigairengyoto Exerting Antibacterial Effects against Staphylococcus aureus
Source: Int J Mol Sci. 2018 Jan 23;19(2):328. doi: 10.3390/ijms19020328 (PMC5855550; doi:10.3390/ijms19020328)
Supplement: Supplementary file 1 [file ijms-19-00328-s001.pdf]

Supplementary

Pharmacokinetic Study of Bioactive Flavonoids in the Traditional Japanese Medicine Keigairengyoto Exerting Antibacterial Effects against *Staphylococcus aureus*

Takashi Matsumoto [1,*], Atsushi Kaneko [1], Junichi Koseki [1], Yosuke Matsubara [1], Setsuya Aiba [2] and Kenshi Yamasaki [2]

[1] Tsumura Kampo Research Laboratories, Kampo Research & Development Division, Tsumura & Co., Ibaraki 300-1192, Japan; kaneko_atsushi@mail.tsumura.co.jp (A.K.); htkj9626m@mineo.jp (J.K.); matsubara_yousuke@mail.tsumura.co.jp (Y.M.)

[2] Department of Dermatology, Tohoku University Graduate School of Medicine, Miyagi 980-8574, Japan; saiba@med.tohoku.ac.jp (S.A.); kyamasaki@med.tohoku.ac.jp (K.Y.)

* Correspondence: matsumoto_takashi@mail.tsumura.co.jp; Tel.: +81-29-889-3852; Fax: +81-29-889-3870

Table S1. Methods of LC-MS/MS: Ion parameters of test compounds.

Compound	Q1 (*m/z*)	Q3 (*m/z*)	DP (volts)	CE (volts)	CXP (volts)	System no.-method no.	Analyzed sample
Glycyrrhizic acid	840.37	453.3	31	43	8	1-1	Plasma_1
Glycyrrhetinic acid	471.234	91	11	111	16	1-1	Plasma_1
Wogonoside	459.174	267.9	-75	-40	-15	1-2	Plasma_1 and plasma_2
Baicalin	447.05	271.1	66	25	18	2-1	Plasma_1
Baicalin	447.05	123	66	77	12	2-1	Plasma_2
Berberine	336.1	320	101	39	10	2-1	Plasma_1 and plasma_2
Hesperetin	300.982	163.8	-140	-34	-23	1-2	Plasma_1 and plasma_2
Luteolin	287.005	153	116	43	14	2-1	Plasma_2
Luteolin	285.058	132.9	-110	-48	-17	1-2	Plasma_1
Wogonin	283.156	268.015	-105	-24	-29	1-2	Plasma_1 and plasma_2
Naringenin	271.235	150.9	-65	-24	-17	1-2	Plasma_1 and plasma_2
Apigenin	271.103	153	111	43	12	2-1	Plasma_2
Baicalein	271.088	123	101	45	10	2-1	Plasma_1 and plasma_2
Apigenin	269.232	116.9	-120	-42	-15	1-2	Plasma_1

Genistein	269.219	132.9	-120	-40	-13	1-2	Plasma_1 and plasma_2
Liquiritigenin	255.037	119	-65	-30	-11	1-2	Plasma_1 and plasma_2
Atropine (IS)	290.019	124.1	111	31	14	1-1	Plasma_1
Niflumic acid (IS)	283.1	265.1	101	31	20	2-1	Plasma_1 and plasma_2
Niflumic acid (IS)	280.826	236.8	-60	-30	-16	1-2	Plasma_1 and plasma_2

[†]: System no.-method no. is linked to number described in Table S2. We used two LC-MS/MS systems in this study as follows: system no. 1, a TripleQuad6500 (AB SCIEX, Tokyo, Japan) equipped with an Agilent 1290 system (Agilent Technologies, Tokyo, Japan); system no. 2, an API4000 triple quadrupole mass spectrometer (AB SCIEX) equipped with an Agilent 1100 system (Agilent Technologies). Plasma_no.: Plasma_1, plasma without β-glucuronidase treatment; plasma_2, plasma with β-glucuronidase treatment. DP; declustering potential, CE; collision energy, CXP; collision cell exit potential.

Table S2. LC-MS/MS Methods: HPLC Conditions.

System no.-method no.	HPLC condition
1-1	Column: CAPCELL CORE ADME (100 × 2.1 mm I.D., 2.7-μm particle size; Shiseido, Tokyo, Japan)<br>Guard column: CORE ADME EXP cartridge (5 × 2.1 mm I.D., 2.7-μm particle size; Shiseido)<br>Mobile phase (A) 10 mmol/L ammonium acetate, (B) methanol<br>Gradient elution program (% B in A):<br>0–1 min, 30%; 1–3 min, 30–50%; 3–10 min, 50–95%; 10–15 min, 95%; 15–15.01 min, 95–30%; 15.01–20 min, 30%<br>Other conditions were: flow rate, 0.3 mL/min; column temperature, 40°C
1-2	Column: Ascentis Express RP-amide column (100 × 2.1 mm I.D., 2.7-μm particle size; Supelco, Bellefonte, PA)<br>Guard column: Ascentis Express RP-Amide guard cartridge (5 × 2.1 mm I.D., 2.7-μm particle size; Supelco)<br>Mobile phase (A) 0.2 vol % acetic acid, (B) acetonitrile containing 0.2 vol % acetic acid<br>Gradient elution program (% B in A):<br>0–5 min, 7%; 5–20 min, 7–95%; 20–20.01 min, 95–7%; 20.01–25 min, 7%<br>Other conditions were: flow rate, 0.35 mL/min; column temperature, 40°C
2-1	Column: Ascentis Express RP-amide column (100 × 2.1 mm I.D., 2.7-μm particle size; Supelco)<br>Guard column: Ascentis Express RP-Amide guard cartridge (5 × 2.1 mm I.D., 2.7-μm particle size; Supelco)<br>Mobile phase (A) 0.2 vol % formic acid, (B) methanol containing 0.2 vol % formic acid<br>Gradient elution program (% B in A):<br>0–5 min, 7%; 5–20 min, 7–95%; 20–20.01 min, 95–7%; 20.01–25 min, 7%<br>Other conditions were: flow rate, 0.35 mL/min; column temperature, 40°C

LC-MS/MS system: system no. 1, a TripleQuad6500 (AB SCIEX) equipped with an Agilent 1290 system (Agilent Technologies); system no. 2, an API4000 triple quadrupole mass spectrometer (AB SCIEX) equipped with an Agilent 1100 system (Agilent Technologies).
